# Genomic surveillance of enterovirus associated with aseptic meningitis cases in southern Spain, 2015–2018

**DOI:** 10.1038/s41598-021-01053-4

**Published:** 2021-11-02

**Authors:** Fabiana Gámbaro, Ana Belén Pérez, Eduardo Agüera, Matthieu Prot, Luis Martínez-Martínez, María Cabrerizo, Etienne Simon-Loriere, Maria Dolores Fernandez-Garcia

**Affiliations:** 1 Institut Pasteur, Paris, France; 2 Hospital Universitario Reina Sofía, Córdoba, Spain; 3 Instituto Maimónides de Investigación Biomédica de Córdoba (IMIBIC), Córdoba, Spain; 4 Universidad de Córdoba, Córdoba, Spain; 5 National Centre for Microbiology, Instituto de Salud Carlos III, Madrid, Spain; 6 CIBER de epidemiología y Salud Pública (CIBERESP), Madrid, Spain; 7 Red de Investigación Translacional en Infectología Pediátrica (RITIP), IdiPaz, Madrid, Spain; 8 Present Address: National Centre for Microbiology, Instituto de Salud Carlos III, Madrid, Spain

**Keywords:** Microbiology, Virology, Metagenomics

## Abstract

New circulating Enterovirus (EV) strains often emerge through recombination. Upsurges of recombinant non-polio enteroviruses (NPEVs) associated with neurologic manifestations such as EVA71 or Echovirus 30 (E30) are a growing public health concern in Europe. Only a few complete genomes of EVs circulating in Spain are available in public databases, making it difficult to address the emergence of recombinant EVs, understand their evolutionary relatedness and the possible implication in human disease. We have used metagenomic (untargeted) NGS to generate full-length EV genomes from CSF samples of EV-positive aseptic meningitis cases in Southern Spain between 2015 and 2018. Our analyses reveal the co-circulation of multiple Enterovirus B (EV-B) types (E6, E11, E13 and E30), including a novel E13 recombinant form. We observed a genetic turnover where emergent lineages (C1 for E6 and I [tentatively proposed in this study] for E30) replaced previous lineages circulating in Spain, some concomitant with outbreaks in other parts of Europe. Metagenomic sequencing provides an effective approach for the analysis of EV genomes directly from PCR-positive CSF samples. The detection of a novel, disease-associated, recombinant form emphasizes the importance of genomic surveillance to monitor spread and evolution of EVs.

## Introduction

Enteroviruses (EVs) belong to the family *Picornaviridae*, genus *Enterovirus*. Their positive single-strand RNA genome is about 7,500 nucleotides (nt), and is composed of a large open reading frame (ORF) flanked by 5’ and 3’ untranslated regions (UTRs)^1^. The 5’ part of the ORF encodes the structural proteins that form the capsid (among them the VP1 which is the most external), while the 3’ part of the ORF encodes the non-structural proteins. There are more than 100 types of EVs infecting humans which are classified into four species A to D^2^. EVs are associated with a wide spectrum of clinical symptoms ranging from nonspecific febrile illness or upper respiratory illness to severe neurological conditions, including meningitis, encephalitis or acute flaccid paralysis^1^.

Genetic recombination is a major process in EVs evolution^3^. Since new recombinant EVs may present different properties (variations in virulence or transmissibility), it is essential to capture complete genomes to detect the occurrence of these recombination events for surveillance and public health purposes. Application of metagenomic next-generation sequencing (mNGS) approaches to characterize the whole-genome sequence of EVs has become a valuable tool for detecting multiple EVs that may be present during co-infection, especially caused by two EV types of same species. Currently, Sanger sequencing of the VP1 capsid protein gene is the gold standard for EV genotyping. But as mNGS becomes increasingly affordable, accessible and cost-effective, sequencing the whole genome of EVs will likely allow improved typing in the near future^4^. Therefore, it is crucial that microbiology reference laboratories progress towards implementation of NGS methods to fully characterize and respond to EVs and publicly share these sequence data^4^. In most countries in Europe, non-polio EV (NPEV) infections are not notifiable and surveillance is mainly passive^5^. Because there is no specific EV surveillance system, surveillance captures primarily EVs detected from hospitalized patients with neurological infections^5^. Identification and full characterization of the EV types involved in these patients are essential to (1) identify EV types associated with neurological infections and estimate better their associated disease burden, (2) monitor the emergence of new EV strains or new recombinant forms, and (3) gain a better understanding of circulating NPEVs^4^.

Despite the common occurrence of EV-associated meningitis cases in Spain^6–8^, there are limited studies defining their molecular epidemiology and addressing the emergence of recombinant EVs. These studies have sequenced either multiple parts of the genome (the complete VP1 and 3Dpol proteins)^9–15^ or the whole genome^16,17^. However, very few full genomes of EVs circulating in Spain apart from EVA71 or EVD68 are available in public databases (Echovirus 30 [n = 5], Coxsackie B2 [n = 1] and Coxsackie A6 [n = 3]) making it difficult to understand the evolutionary relatedness of the different EV types and the possible implication in human disease.

Here, we aimed to characterize the genetic diversity of EVs circulating in Southern Spain during 2015–2018. We performed mNGS on 12 CSF samples of laboratory-diagnosed enteroviral meningitis. Our viral genomic analysis revealed the co-circulation of E6, E11, E13 and E30 types including a novel E13 recombinant form.

## Results

### Clinical, epidemiological and laboratory features of patients with enteroviral meningitis

From 2015 to 2018, twelve adult patients were laboratory-diagnosed with enteroviral meningitis at the University Hospital Reina Sofia (Córdoba, Spain). All samples were identified as EVs positive but no other typing data was available for these strains. In this work, we investigated the twelve CSF samples collected from these patients by using ribosomal RNA depletion before mNGS on total extracted RNAs. Epidemiological and clinical information about each patient is listed in Table 1. The median age of the patients was 27.6 years (range 17–36 years) with a female-to-male ratio of 1:0.7. All patients were living in the Cordoba Province, located in southern Spain. Cases showed a seasonal pattern, with E6, E13 and E30 infections occurring during spring (March–May) months, while E11 cases occurred during winter (November and December). The most common symptoms in patients were headache (100%) and fever (90.9%). Seven patients (58.3%) reported contact with potential EV-infected children at home that presented fever or gastrointestinal symptoms. All patients had pleocytosis, defined as CSF WBC counts of > 5/mm^3^, with a median WBC count of 286/mm^3^ and predominance of lymphocytes (from 42 to 91% of WBC) (Table 1).

**Table 1:** Clinical features and laboratory findings for the cases of enteroviral meningitis, Córdoba, Spain, 2015–2018.

EV type	Sample Nº	Age (y)	Sex	Clinical features	Date sample collection	Days of hospitalization	CSF analysis			
							WBC mm^3^	Lymph %	Gluco (mg/dl)	Prot (mg/dl)
E6	138	19	F	Fever, headache, vomiting, neck stiffness, GI	May-2015	2	300	48	94	41
	255	31	M	Fever, headache, nausea, GI	Apr-2015	4	233	42	59	69
	268	27	F	Fever, headache, nausea, vomiting	Apr-2015	6	118	63	55	46
	365	23	F	Fever, headache	May-2015	2	203	61	63	41
E11	484	29	F	Fever, headache, nausea	Nov-2015	2	86	90	53	47
	1059	29	M	Fever, headache, photophobia, GI	Dic-2018	3	383	72	NA	68
	1106	36	F	Fever, headache	Dic-2018	2	312	90	55	63
E13	53	35	F	Fever, headache, nausea, GI	May-2016	10	812	72	50	113
E30	265	30	F	Fever, headache, neck stiffness	Feb-2016	1	101	90	53	45
	519	31	M	Fever, headache, vomiting	Mar-2018	2	422	91	NA	63
	520	25	M	Headache, vomiting, photophobia, GI	May-2018	4	310	82	NA	41
	675	17	M	Fever, headache, vomiting, photophobia	Mar-2017	3	156	63	60	58

*EV* enterovirus, *CSF* cerebrospinal fluid, *GI* gastrointestinal symptoms, *Gluco* glucose, *NA* not available, *Prot* proteins, *WBC* White Blood Cells.

### Complete genome analysis

After quality trimming, we assembled the reads using MetaSPAdes v3.12, and used Diamond to query the contigs against the non-redundant protein database (NCBI). For each sample, we obtained a large contig corresponding to a near-complete enterovirus genome, or fragments that could be assembled into a genomic scaffold. We did not detect co-infection with other pathogens, including those that have been found in the CSF of patients with meningoencephalitis such as Dengue virus, Chikungunya virus or West Nile virus^18^. Results of the metagenomic analysis can be found in Table 2. De novo assembly resulted in the reconstruction of complete or near-complete EVs genome for all 12 samples. All viruses presented high sequence coverage throughout the genome as shown in Supplementary Fig. S1. Overall, 4 different EV types belonging to species-B were identified. Four samples were found to contain E6, three samples E11, four samples E30 and one sample E13. Genomes ranged from 7213 to 7451 nt in length, encoding polyproteins of 2188, 2191, 2194 and 2195 aa for E13, E6, E30 and E11, respectively.

**Table 2:** RNA-sequencing results using mNGS from CSF samples.

	Sample Nº	Number of mapped viral reads	Mapped viral reads (%)	Genome covered	Average coverage depth
E6	138	127,810	1.2	99.30%	1273
	255	70,030	0.6	99.88%	697
	268	56,357	0.59	99.86%	560
	365	6524	0.14	99.31%	64
E11	484	1046	0.01	95,76%	10
	1059	240,901	2.76	99.40%	2383
	1106	366,776	3.13	99.70%	3624
E13	53	159,567	1.61	100%	1593
E30	265	68,949	0.35	98.91%	684
	519	54,571	0.33	99.30%	537
	520	628,623	4.39	99.60%	6214
	675	91,887	0.76	99.50%	909

### Phylogenetic analysis

**Figure 1:**
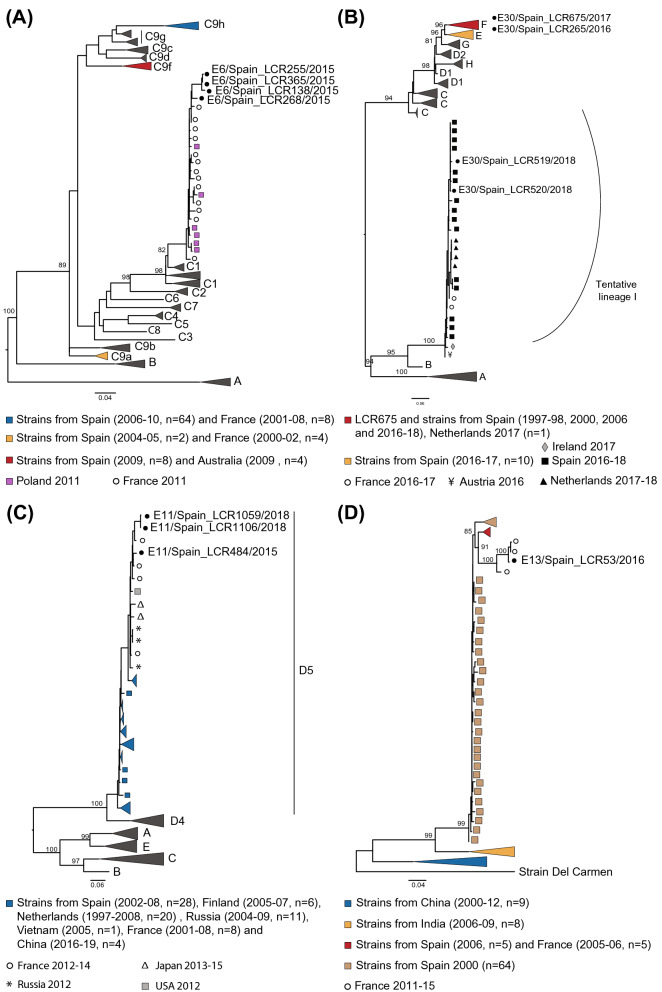
Maximum-likelihood trees based on a partial VP1 inferred by IQ-TREE v.2.0.6 for the four genotypes study here: E6 (**A**), E30 (**B**), E11 (**C**) and E13 (**D**). Sequences reported in our study are indicated by black circles. Numbers on nodes indicate the bootstrap support of the node (> 80) and the scale bars represents the expected number of nucleotide substitutions per site. Non collapsed trees can be found in Supplementary Fig. 4A–D. Trees are annotated with the classification proposed by Smura et al. and Cabrerizo et al.^10,19^ for E6, the classification proposed by Bailly et al.^20^ for E30, and the classification proposed by Li et al.^22^ for E11. Only bootstrap values above 80% are indicated in branch nodes. Scale bars indicate nucleotide substitutions per site.

In order to characterize the study strains and evaluate their evolutionary relationship to previously characterized homotypic strains detected in Spain and globally in the last decades, phylogenetic analyses were performed for the four encountered genotypes first using the VP1 region (Fig. 1).

E6 lineages were assigned according to Smura et al. and Cabrerizo et al. into lineages A-C^10,19^. Phylogenetic analysis showed that all E6 strains detected in 2015 clustered within sublineage C1 (bootstrap value of 97%). Previously reported E6 strains from Spain (detected between 2004 and 2010) fell into the sublineage C9, clustering into three distinct groups (C9a, C9f. and C9h) (Fig. 1A). All four E6 study strains showed together high nucleotide similarity in the complete VP1 region (mean 97.8% (96.2–99.2%) nt and 99.2% (98.6–100%) aa identity) and branched together within the diversity of strains detected in France and Poland in 2011.

E30 lineages were defined according to Bailly et al. into lineages A-H^20^. The E30 study strains clustered in two different lineages. Sequences LCR265 (2016) and LCR675 (2017) clustered within lineage F, a group that contained most of E30 strains previously identified in Spain (from 1996 to 2018). Analysis with complete VP1 sequences (Supplementary Fig. S2) showed that while LCR675 strain forms a subcluster (bootstrap value of 100%) with other E30 strains from Spain isolated in 2016 in Catalonia (displaying identities of at least 96.4% and 91.1% at nucleotide and peptide levels, respectively), LCR265 cluster with viruses detected in France, Luxembourg and the Netherlands in 2016–2017 suggesting the circulation of two different sublineages. In contrast, strains LCR519 and LCR520 from 2018 grouped into a distinguishable lineage, tentatively called here “lineage I” (corresponding to clade G6 in the classification of Benschop et al.^21^ which contains sequences from Netherlands, France, Ireland and Spain, sampled in the same time period (2017–2018) (Fig. 1B). Similar pattern of clustering was obtained when the complete VP1 region was used for phylogenetic inference (Supplementary Fig. S2). A notable observation is that “lineage I” is more closely related to lineage B, which was not detected beyond the 1970s^20^ (mean *p*-distance of 0.17), than to more recent lineages like E and F which contain worldwide E30 strains including all E30 strains previously reported in Spain (mean *p-*distance between lineage I and lineages E and F of 0.25 and 0.26, respectively) (Supplementary Table S1).

All E11 strains obtained in this study belong to subgenotype D5 according to the classification introduced by Li et al.^22^ which included stains detected in Europe and Asia that have circulated for over 20 years (Fig. 1C). E11 study strains were all highly similar (nt identity ≥ 95.3%; aa identity ≥ 97.6%) and were closely related to strains obtained in France in 2014 from meningitis cases in children (mean 96.5% (95.7–97.8%) nt and 98.6% (98.2–99.3%) aa similarities in the complete VP1 region).

Finally, Fig. 1D shows that E13 study strain LCR53 from 2016 cluster closely (bootstrap value of 100%) with two strains collected from children with meningitis in France in 2015 (nt identity ≥ 98.8%; aa identity ≥ 99.6%).

### Recombination analysis

**Figure 2:**
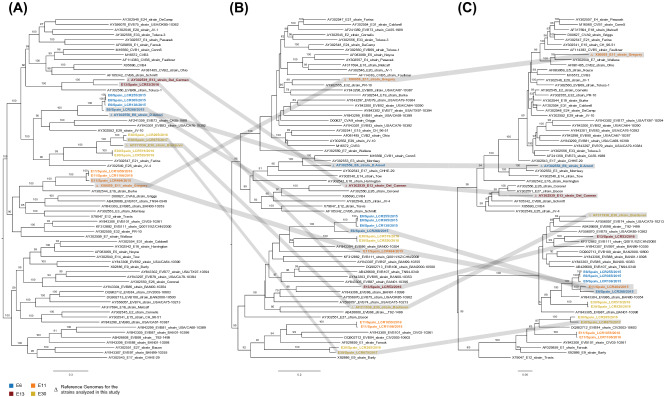
Phylogenetic analysis based on the P1, P2 and P3 coding sequences of the 12 study strains and other fully sequenced EV-B global strains. Maximum-likelihood phylogenetic trees were inferred using IQ-TREE2 on an alignment of ~ 2565, 1734 and 2268 nt sequences corresponding to the P1 (**A**), P2 (**B**), and P3 (**C**) coding regions respectively. The numbers at the nodes indicate bootstrap support values > 80 for that node. Scale bar represents nucleotide substitutions per site. GenBank accession numbers for published sequences are shown in the tree.

Considering the prevalence of recombination in the evolutionary history of enteroviruses, we next looked for evidence of recombination in the study strains. Recombination analyses were carried out using a combination of six methods implemented in RDP5.5^23^. We detected multiple events of recombination in the evolutionary history of the EV types studied. This was also evidenced when comparing the topologies of phylogenies build using the 3 coding regions (P1, P2 and P3) of the sequences from this study and representative EV-B reference genomes available in GenBank (Fig. 2). The phylogenetic analysis using the P1 capsid coding region showed that the E6, E11, E13 and E30 sequences obtained from this study cluster together with their respective reference genome which coincides with what we observed using the partial VP1 region (Fig. 2A). However, this was no longer observed when constructing the phylogeny based on the P2 and P3 non-capsid genomic regions (Fig. 2B,C). These incongruent tree topologies between the capsid and non-capsid regions indicate that genetic exchanges through intertypic recombination with other EV-B types might have occurred. We also used similarity plot and bootscanning analyses comparing the study strains and closely related types to visualize the mosaic genomes reported here. The sequences of closely related types included in the analysis were those with highest score when using the genomic regions identified in the RDP5.5 analysis as of recombinant origin as queries for BLASTn (http://www.ncbi.nlm.nih.gov/) (Supplementary Table S2).

Genome sequences of E11 study strains were compared with all E6 study strains, the E11 prototype strain and sequences closely related to different parts of LCR484 which was identified as mosaic by all 6 methods within RDP5.5. While most of LCR484 genome presented high identity with the other E11 strains reported here (LCR1106, LCR1059), the 3’ end of the genome (from the 3C region) presented high identity with non-E11 sequences. Genomes with similar recombinant genomic organization have been detected previously (Supplementary Fig. S3).

**Figure 3:**
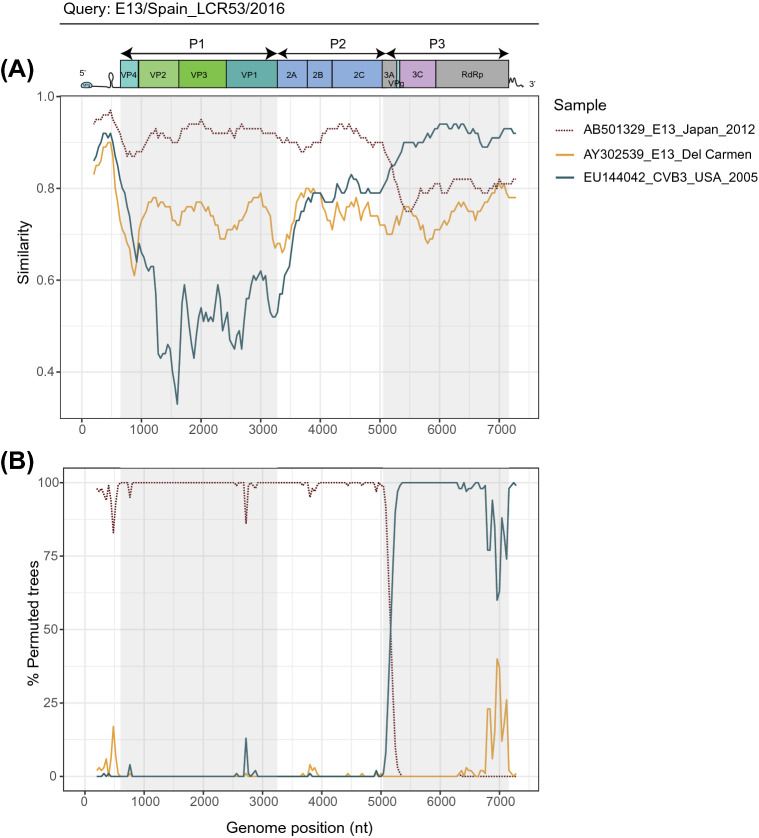
Plot of similarity (**A**) and bootscanning analysis (**B**) of E13/Spain_LCR53/2016 study strain with prototype and closely related strains. The enterovirus genetic organization is shown in the top panel. Analyses were conducted by using SimPlot 3.5.1 (Kimura distance model, window size 400 bp moving in 40 nucleotides steps).

The LCR53 (E13) genome also presented strong signal of recombination, both intra and intertypic. In particular, we noted signal of recombination with other E13 strains in the 5’ (P1 and P2 regions) of the genome. Most importantly, we detected intertypic recombination signal in the 3’end of the genome (from the 3C region) (Fig. 3). No similar recombinant genomes have been reported.

Among the E30 strains reported here, those from lineage I (LCR 519 and LCR520) and those from lineage F (LCR675 and LCR265) were detected as of recombinant origin, with signal in the 3’end of the genome (Supplementary Fig. S3). However, like for E11, genomes with similar mosaicism have been reported in the Netherland during the same year (Supplementary Fig. S3).

## Discussion

Application of mNGS methods to characterize the whole-genome sequence of viruses has become a valuable tool for tracking viral evolution, to elucidate the mechanisms shaping their diversity and to better understand the biology of viruses associated with human disease^24^. In the present study, we used a previously described^25^ protocol for mNGS of clinical samples on twelve CSF samples confirmed as EV-positive and collected from patients with aseptic meningitis during 2015–2018 in Southern Spain. It is important to note that the clinical samples were previously frozen and thawed at least once and stored at − 45 °C. Our approach allowed to obtain complete or near-genomes despite the length of storage (samples were collected from 2015–2018 and processed in 2019), freeze–thaw cycles and the storage temperature above − 480 °C. This suggests that this method can be effectively implemented in public health reference laboratories to detect and reconstruct complete viral genomes from CSF samples even if there has been a progressive degradation of RNAs. Moreover, unlike PCR amplicon-based or capture-based enrichment methods, this metagenomic approach does not require previous knowledge of the pathogen sequence, making this approach suitable to characterize novel viruses or recombinant forms. This is important as upsurges of emerging recombinant NPEVs associated with neurologic manifestations such as EVA71 or E30 are a growing public health concern in Europe^26,27^. A real-time detection and rapid sharing of whole-genome sequence information on these emerging NPEVs from public health reference laboratories may help detect outbreaks of severe neurologic disease and implement early intervention strategies ensuring rapid control of disease spread.

The types identified in this study were E6, E11, E13 and E30. Echoviruses belong to the EV-B species and constitute the largest group of the EV genus, with 28 types. A meta-analysis of the worldwide distribution of EVs showed that E30, E6 and E13 were the most commonly detected human EV types in meningitis cases^28^. In Europe, between 2015 and 2017, E30, E6 and E11 were among the ten most frequently reported types representing 12%, 12% and 4%, respectively, of all typed EV-positive samples^5^. In 2018, an E30 upsurge was observed in Denmark, Germany, The Netherlands, Norway and Sweden associated with meningitis or meningoencephalitis^26^. A recent study identified that this upsurge was caused by co-circulation of E30 viruses from two different lineages: E and I (corresponding to clades G1 and G6, respectively) that replaced E30 viruses from lineage F (corresponding to clade G2) which predominated in 2016 and 2017^21^. We similarly note that the E30 strains detected in Spain in 2017 are from lineage F, and that the subsequent strains (from 2018) correspond to the emerging lineage I. The close genetic relatedness of the E30 sequences reported in lineage I during 2017–2018 in the Netherlands, France, Ireland and Spain is further indicative of rapid widespread transmission of this lineage^29^.

In Spain, from 1988 to 2008, E30 has been the predominant EV type (33.7% of the total typed EVs)^8,30^. However, in 2009, an upsurge of E6 (60%) replaced E30 as the predominant type. In more recent years (2016–2019), E30 and E6 were among the ten most prevalent types of all typed EVs in Spain^6,31^. All E6 study strains analyzed in this study were from 2015 and segregated into the same sublineage C1, together with previously described strains from France and Poland isolated in 2011, indicating that related C1 strains were circulating during those years over a broad geographic area in Europe. Interestingly, E6 strains circulating in Spain (n = 86) in the previous decade (2000–2010), all clustered into sublineage C9 suggesting again, as was observed for E30, a genetic turnover where C9 viruses might have subsequently be replaced by C1 viruses.

In this study, phylogenetic analysis showed that E11 study strains from 2015 and from 2018 belong to the same genomic cluster (subgenotype D5) which is distributed worldwide. Study strains have close relationship with each other (> 95% identity) suggesting sustained local circulation in the region during the study period. The fact that strains detected in Spain from 2002 to 2008 also cluster in D5 supports the idea that this subgenotype has persisted for almost two decades in the country and is in agreement with previous studies describing relative genetic stability of E11 strains, associated with long-term endemic patterns^13,32^. Of note, although E11 study strains grouped within the previously described D5 cluster, they formed a separate subcluster suggesting genetic evolution of this endemic E11 viral population from a common ancestor.

Consistently with Europe where E13 is among the less reported types (< 1%), E13 is also rarely reported in Spain^5,6,33^. It appears to circulate in a cyclical fashion with epidemic years followed by years with low detection rates^33^. In Spain, E13 reporting increased in 2000 and 2019 (45% and 8% of the total typed EVs, respectively) with almost no detection in between^6,33^. In this study, we detected a sporadic case of aseptic meningitis associated with an E13 strain from 2016 closely related to E13 strains from France also collected in 2016 from children with meningitis. This close genetic relatedness between E13 sequences reported in 2016 in both France and Spain suggests rapid widespread transmission.

Recombination is known to constitute a widespread mechanism of EV evolution, through the continual exchange of fragments between co-circulating viruses which may impact viral replication and pathogenicity. Full genome sequencing is crucial to detect these recombination events. Our analysis revealed that the E6, E11, E13 and E30 circulating types present complex mosaic genomes constituted of fragments of different origins, implicating intertypic recombination events.

Our analysis highlights the continued circulation of recombinant E11 viruses carrying non-structural sequences similar to other (non-E11) types. Evidence of evolution of E6 and E11 through intertypic recombination has been suggested before^34^, and the detection of strains with similar mosaic organization in multiple parts of the world (USA, Japan, Italy) indicates that this lineage has been circulating worldwide in recent years, in parallel to other E11 lineages.

Within E30, the lineage F strains reported here (LCR265, 2016 and LCR675, 2017) present a mosaic pattern previously detected in genomes from Spain (Catalonia) in 2016, as well as genomes noted in the Netherlands in 2017. In a similar way, the lineage I study strains (LCR519 and LCR520, 2018) also present mosaic patterns previously detected in genomes from the Netherlands and Ireland in 2017 and from Austria in 2016. These results suggest that these recombinant lineages have been circulating for some time and started to diversify. Multiple reports highlight the involvement of genetic recombination in the evolutionary process of E30 with other EV-B strains in the non-structural region^13,29,35^.

The E13 study strain (LCR53) presents a mosaic organization with no reported homologs, suggesting that the recombination event leading to the circulation of this virus might be recent, or that it has been circulating at low noise and was not captured up to now. The detected recombination breakpoint lies at the beginning of the P3 region, providing further support to the idea that the structural and non-structural genome regions of EV-B types evolve independantly^36^. To better monitor recombination events and more accurately identify potential recombination partners or location of emergence (including potential geographical hotspots), stronger genomic surveillance programs for EVs are needed.

In Europe, EVs are more commonly detected in late summer and autumn^5^. Our study revealed that most types (E6, E13 and E30) were detected during the spring season (March–May) which is consistent with previous reports describing the incidence of EV-B in Spain peaking during spring^8,30,37^. Rather unexpectedly, all three cases associated to E11 occurred in winter. Different seasonality in circulating EV types and the fact that seasonality in temperate zones such as Spain is less marked than in tropical climates could explain these differences.

One limitation of this study is the limited number of samples here analyzed within a 3-year period (only severe cases requiring hospitalization). For this reason, we are likely underestimating the genetic diversity of EVs in Southern Spain. This emphasizes the need to improve the genomic surveillance of EVs in Spain by screening larger cohorts of patients and obtaining detailed epidemiological and clinical data. This could be complemented with increased nationwide surveillance and further exploration of environmental samples using mNGS to monitor emergent and/or novel recombinant types and to better understand their circulation patterns and potential recombination events among co-circulating EVs.

In conclusion, this study documents for the first time the whole-genome sequences of E6, E11 and E13, as well as E30 circulating in Spain. The detection of a novel, disease-associated, and recombinant form highlights the importance of genomic surveillance characterizing full-length EV genomes and public sharing of whole-genome sequence data.

## Materials and methods

### Ethics statement

Ethical approval for the experimental protocol was given by the Research Ethics Committee from Cordoba (Comité de Ética de Investigación de Córdoba, reference 201999903552445). The procedures were carried out in accordance with approved guidelines, regulations and the principles of the Declaration of Helsinki. Written informed consent was obtained from all patients or from a parent and/or legal guardian.

### Study background

From January 2015 to December 2018, eleven adult patients were diagnosed in the Department of Neurology in the University Hospital Reina Sofia (Córdoba, Spain) with a laboratory-confirmed enteroviral meningitis. The Department of Neurology is responsible for the treatment of patients from 14 years old and is the reference unit for patients with suspected CNS infections in the province. The University Hospital Reina Sofia is a 1280-bed tertiary referral hospital for the southwestern Spanish province of Córdoba (population 461.078). A case was defined as meningitis-suspected based upon the presence of these symptoms: fever, headache, vomiting, neck stiffness, nausea and sometimes accompanied by photophobia, abdominal pain or diarrhea. CSF samples were obtained at patient admission according to standard procedures and analyzed at the Department of Microbiology. Samples were tested for the presence of EVs using a qualitative multiplexed PCR (FilmArray^®^ Meningitis/Encephalitis Panel, BioFire Diagnostics) or RT-PCR (Xpert EV^®^, Cepheid). In all CSF samples, bacterial, herpes simplex virus types 1 and 2, and varicella-zoster virus infections of the CNS were excluded by culture or PCR. Bacterial screening was performed by culture on chocolate agar, blood agar and thioglycollate broth, while herpes simplex virus and varicella zoster virus screening was performed by a qualitative multiplexed PCR (FilmArray^®^ Meningitis/Encephalitis Panel, BioFire Diagnostics) or RT-PCR (RealCycler^®^ herpesvirus type 1 + herpesvirus type 2 + varicella-zoster virus, Progenie Molecular). Sample remnants were stored at − 45 °C. Clinical, epidemiological and laboratory data were collected retrospectively from medical records.

### Library preparation and metagenomic sequencing

RNA was extracted from 140 μl of CSF using the QIAamp Viral RNA Mini Kit (Qiagen) followed by Turbo DNAse treatment (Ambion) and purification with Agencourt RNAClean XP beads. We next depleted host rRNA using custom probes and RNAse H treatment as previously described^25^. Depleted samples were purified using AMPure RNA clean beads (Beckman Coulter Genomics) and eluted in 10 ul of RNase-free water for cDNA synthesis. RNA was converted to double-stranded cDNA in two steps. First, RNA was retro transcribed using random primers and SuperScript IV (Invitrogen). Second-strand cDNA was generated using E. coli DNA ligase, RNAse H and DNA polymerase (New England Biolabs) and purified using Agencourt AMPure XP beads (Beckman Coulter). Libraries were then prepared using the Nextera XT DNA Library Prep Kit (Illumina) and sequenced on an Illumina NextSeq500 (2 × 75 cycles).

### Genome assembly and analysis of sequence data

Raw reads were trimmed using Trimmomatic v0.36 to remove low-quality reads and Illumina adaptors. Reads were de novo assembled using the metaspades option from SPAdes v3.12 and the contigs obtained were used as blast queries on Virus Pathogen Resource (ViPR)^38^. We used clc-assembly-cell v5.1.0 to perform mapping and extract consensus. A minimum of 3X read depth coverage was used, and N were added in case of lower coverage. Samtools v1.3 was used to sort the aligned BAM files and to generate alignment statistics. All alignments and consensus sequences were manually inspected using Geneious Prime 2020.2 (https://www.geneious.com/).

### Recombination analysis

Recombination analyses were carried out on the study strain using a background of randomly sampled EV genomes retrieved from GenBank on May 2021, using a combination of six methods implemented in RDP5.5^23^ (RDP, GENECONV, MaxChi, Bootscan, SisScan and 3SEQ) and we considered recombination signals detected by more than three methods for breakpoint identification. Except for specifying that sequences are linear, all settings were kept to their defaults. In addition, similarity plot and bootscanning analysis were performed by using the SimPlot program, version 3.5.1, with a 400-nt window moving in 40-nt steps and using a Kimura 2-parameter method with a transitions-transversions ratio of 2 with 1000 resampling.

### Phylogenetic analysis

We performed phylogenetic analysis of the 12 genomes from our dataset, together with published genomes available on NCBI GenBank retrieved on May 2021. Because the majority of the reported sequences from strains circulating in Spain consisted of partial VP1 sequences, we first performed phylogenetic analysis based on the alignment of partial 3’-half VP1 region (E6, E30, and E13) or in the 5’-half VP1 region (E11). Nt sequence alignment was performed by using ClustalW multiple alignment program^39^ within the BioEdit sequence Alignment Editor package, version 7.0.9.0. Maximum-likelihood trees were constructed using IQ-TREE2 (v.2.0.6)^40^ with 1000 bootstrap replicates^41^. We constructed the trees using the best-fit model as determined by ModelFinder^42^ implemented in IQ-TREE2. Genetic distances and sequence divergence were calculated using the Molecular Evolutionary Genetics Analysis (MEGA-X) software package (http://megasoftware.net/).

## Supplementary Information

Supplementary Information.

Supplementary Figure S1.

Supplementary Figure S2.

Supplementary Figure S3.

Supplementary Figure S4.

Supplementary Figure S4.

Supplementary Figure S4.

Supplementary Figure S4.

## Data Availability

Raw sequencing reads were deposited on the European Nucleotide Archive (https://www.ebi.ac.uk/ena/browser/home) with study number PRJEB45068. The GenBank (https://www.ncbi.nlm.nih.gov/genbank/) accession numbers of the assembled virus genomes are MZ389224—MZ389234 and MZ436966. Both ENA and GenBank are part of the International Nucleotide Sequence Database Collaboration (INSDC https://www.insdc.org/).
