# Author Correction: Genomic surveillance of enterovirus associated with aseptic meningitis cases in southern Spain, 2015–2018

**DOI:** 10.1038/s41598-021-02194-2

**Published:** 2021-11-18

**Authors:** Fabiana Gámbaro, Ana Belén Pérez, Eduardo Agüera, Matthieu Prot, Luis Martínez-Martínez, María Cabrerizo, Etienne Simon-Loriere, Maria Dolores Fernandez-Garcia

**Affiliations:** 1 Institut Pasteur, Paris, France; 2 Hospital Universitario Reina Sofía, Córdoba, Spain; 3 Instituto Maimónides de Investigación Biomédica de Córdoba (IMIBIC), Córdoba, Spain; 4 Universidad de Córdoba, Córdoba, Spain; 5 National Centre for Microbiology, Instituto de Salud Carlos III, Madrid, Spain; 6 CIBER de Epidemiología Y Salud Pública (CIBERESP), Madrid, Spain; 7 Red de Investigación Translacional en Infectología Pediátrica (RITIP), IdiPaz, Madrid, Spain; 8 Present Address: National Centre for Microbiology, Instituto de Salud Carlos III, Madrid, Spain

Correction to: *Scientific Reports* 10.1038/s41598-021-01053-4, published online 02 November 2021

In the original version of this Article Etienne Simon-Loriere and Maria Dolores Fernandez-Garcia were omitted as jointly supervising authors.

The original Article has been corrected.

