# Supplementary material for: Genomic surveillance of enterovirus associated with aseptic meningitis cases in southern Spain, 2015–2018
Source: Sci Rep. 2021 Nov 2;11:21523. doi: 10.1038/s41598-021-01053-4 (PMC8564535; doi:10.1038/s41598-021-01053-4)
Supplement: Supplementary file 1 — Supplementary Information. [file 41598_2021_1053_MOESM1_ESM.docx]

# Supplementary information

Supplementary Figure S1. Genome coverage of Enterovirus study strain genomes by deep sequencing.

**Supplementary Figure S2.** Maximum-likelihood tree based on complete VP1 inferred using IQ-TREE2 for E30.

**Supplementary Figure S3.**  Simplots of E11/Spain_LCR484/2015 (A), E30/Spain_LCR520/2018 (B), E30/Spain_LCR675/2017 (C) and E30/Spain_LCR265/2016 (D) strains against the prototype and closely related sequences. The analyses were performed with SimPlot v3.5.1 using Kimura 2 parameter distance model with a window size of 400 bp and a step size of 40 bp.

**Supplementary Figure S4.** Maximum-likelihood trees based on a partial VP1 for the four genotypes study here: E6 (A), E30 (B), E11(C) and E13 (D) (non-collapsed version of Fig 1).

**Supplementary table S1.** Mean *p*-distance between different E30 lineages.

|  | **Tentative lineage I** | **A** | **B** | **C** | **D** | **E** | **F** | **G** | **H** |
| --- | --- | --- | --- | --- | --- | --- | --- | --- | --- |
| **Tentative lineage I** |  |  |  |  |  |  |  |  |  |
| **A** | 0,28 |  |  |  |  |  |  |  |  |
| **B** | 0,17 | 0,27 |  |  |  |  |  |  |  |
| **C** | 0,24 | 0,29 | 0,23 |  |  |  |  |  |  |
| **D** | 0,25 | 0,30 | 0,24 | 0,16 |  |  |  |  |  |
| **E** | 0,26 | 0,29 | 0,24 | 0,18 | 0,12 |  |  |  |  |
| **F** | 0,25 | 0,29 | 0,24 | 0,18 | 0,12 | 0,11 |  |  |  |
| **G** | 0,26 | 0,29 | 0,25 | 0,18 | 0,12 | 0,13 | 0,14 |  |  |
| **H** | 0,24 | 0,29 | 0,23 | 0,19 | 0,13 | 0,15 | 0,15 | 0,15 |  |

**Supplementary table S2.** Closely related sequences available in the GenBank database selected using BLAST.

| Region | Sample Nº_Type | Closely related type | Strain | Country | Year | Accesion Number | Query cover | Per. Ident |
| --- | --- | --- | --- | --- | --- | --- | --- | --- |
| P3 | LCR53_E13 | CVB3 | CVB3-MCH | USA | 2005 | EU144042 | 100% | 91.37% |
|  |  | E30 | 2002-59 | China | 2002 | KP266571 | 100% | 89.55% |
|  | LCR484_E11 | E11 | ISO_VR | Italy | 2013 | KX527626 | 96% | 94.01% |
|  |  | E11 | 10S1 | USA | 2013 | MN749159 | 96% | 93.93% |
|  | LCR1059_E11 | E25 | 19521 | USA | 2016 | MT347976 | 100% | 86.33% |
|  |  | E20 | CMRHP45 | Cameroon | 2014 | MH933855 | 99% | 86.24% |
|  | LCR1106_E11 | E25 | 19521 | USA | 2016 | MT347976 | 100% | 86.38% |
|  |  | E20 | CMRHP45 | Cameroon | 2014 | MH933855 | 99% | 86.37% |
|  | LCR519_E30 | E30 | NL/17-499 | Netherlands | 2017 | MK815082 | 100% | 98.9% |
|  |  | E30 | NL/17-784 | Netherlands | 2017 | MK815083 | 100% | 98.85% |
|  | LCR520_E30 | E30 | NL/17-499 | Netherlands | 2017 | MK815082 | 100% | 98.63% |
|  |  | E30 | NL/17-784 | Netherlands | 2017 | MK815083 | 100% | 98.59% |
|  | LCR675_E30 | E30 | 18L | Spain | 2016 | MH484073 | 100% | 99.03% |
|  |  | E30 | 20L | Spain | 2016 | MH484075 | 100% | 97.84% |
|  | LCR265_E30 | E30 | NL/16-439 | Netherlands | 2017 | MK815079 | 100% | 98.28 |
|  |  | E30 | NL/16-443 | Netherlands | 2016 | MK815074 | 100% | 98.49% |
|  | LCR138_E6 | E11 | ISO_VR | Italy | 2013 | KX527626 | 100% | 92.77% |
|  |  | E11 | 10S1 | USA | 2013 | MN749159 | 100% | 92.59% |
|  | LCR255_E6 | E11 | ISO_VR | Italy | 2013 | KX527626 | 100% | 92.77% |
|  |  | E11 | 10S1 | USA | 2013 | MN749159 | 100% | 92.59% |
|  | LCR268_E6 | CVB1 | B1L071615 | Switzerland | 2015 | MG845887 | 100% | 94.84% |
|  |  | E11 | 10S1 | USA | 2013 | MN749159 | 100% | 93.69% |
|  | LCR365_E6 | E11 | ISO_VR | Italy | 2013 | KX527626 | 100% | 92.68% |
|  |  | E11 | 10S1 | USA | 2013 | MN749159 | 100% | 92.5% |
| P2 | LCR53_E13 | E13 | 240SF | Japan | 2002 | AB501329 | 100% | 91.46% |
|  |  | E13 | S3(1)-1 | Japan | 2002 | AB501332 | 100% | 91.41% |
|  | LCR484_E11 | E11 | 10S1 | USA | 2013 | MN749159 | 100% | 96.6% |
|  |  | E11 | ISO_VR | Italy | 2013 | KX527626 | 100% | 95.73% |
|  | LCR1059_E11 | E11 | 10S1 | USA | 2013 | MN749159 | 100% | 84.61% |
|  |  | E11 | 34-1-1/GD.GZ | China | 2018 | MN597950 | 100% | 84.61% |
|  | LCR1106_E11 | E11 | 10S1 | USA | 2013 | MN749159 | 100% | 84.61% |
|  |  | E11 | 34-1-1/GD.GZ | China | 2018 | MN597950 | 100% | 84.44% |
|  | LCR519_E30 | E30 | NL/17-784 | Netherlands | 2017 | MK815083 | 100% | 98.73% |
|  |  | E30 | NL/17-499 | Netherlands | 2017 | MK815082 | 100% | 98.73% |
|  | LCR520_E30 | E30 | NL/17-784 | Netherlands | 2017 | MK815083 | 100% | 98.62% |
|  |  | E30 | NL/17-499 | Netherlands | 2017 | MK815082 | 100% | 98.62% |
|  | LCR675_E30 | E30 | 18L | Spain | 2016 | MH484073 | 100% | 99.02% |
|  |  | E30 | 20L | Spain | 2016 | MH484075 | 100% | 97.75% |
|  | LCR265_E30 | E30 | NL/16-443 | Netherlands | 2016 | MK815074 | 100% | 98.62% |
|  |  | E30 | NL/16-302 | Netherlands | 2016 | MK815072 | 100% | 98.50% |
|  | LCR138_E6 | E6 | PMKA1204 | Thailand | 2011 | KU574626 | 100% | 92.62% |
|  |  | E20 | NGR_I011 | Nigeria | 2010 | MN181513 | 98% | 84.39% |
|  | LCR255_E6 | E6 | PMKA1204 | Thailand | 2011 | KU574626 | 100% | 92.85% |
|  |  | E20 | NGR_I011 | Nigeria | 2010 | MN181513 | 98% | 84.62% |
|  | LCR268_E6 | E6 | PMKA1204 | Thailand | 2011 | KU574626 | 99% | 92.32% |
|  |  | E11 | 10S1 | USA | 2013 | MN749159 | 100% | 84.65% |
|  | LCR365_E6 | E6 | PMKA1204 | Thailand | 2011 | KU574626 | 100% | 92.91% |
|  |  | E20 | NGR_I011 | Nigeria | 2010 | MN181513 | 98% | 84.62% |
