# Supplementary figures and images for: Genomic surveillance of enterovirus associated with aseptic meningitis cases in southern Spain, 2015–2018
Source: Sci Rep. 2021 Nov 2;11:21523. doi: 10.1038/s41598-021-01053-4 (PMC8564535; doi:10.1038/s41598-021-01053-4)

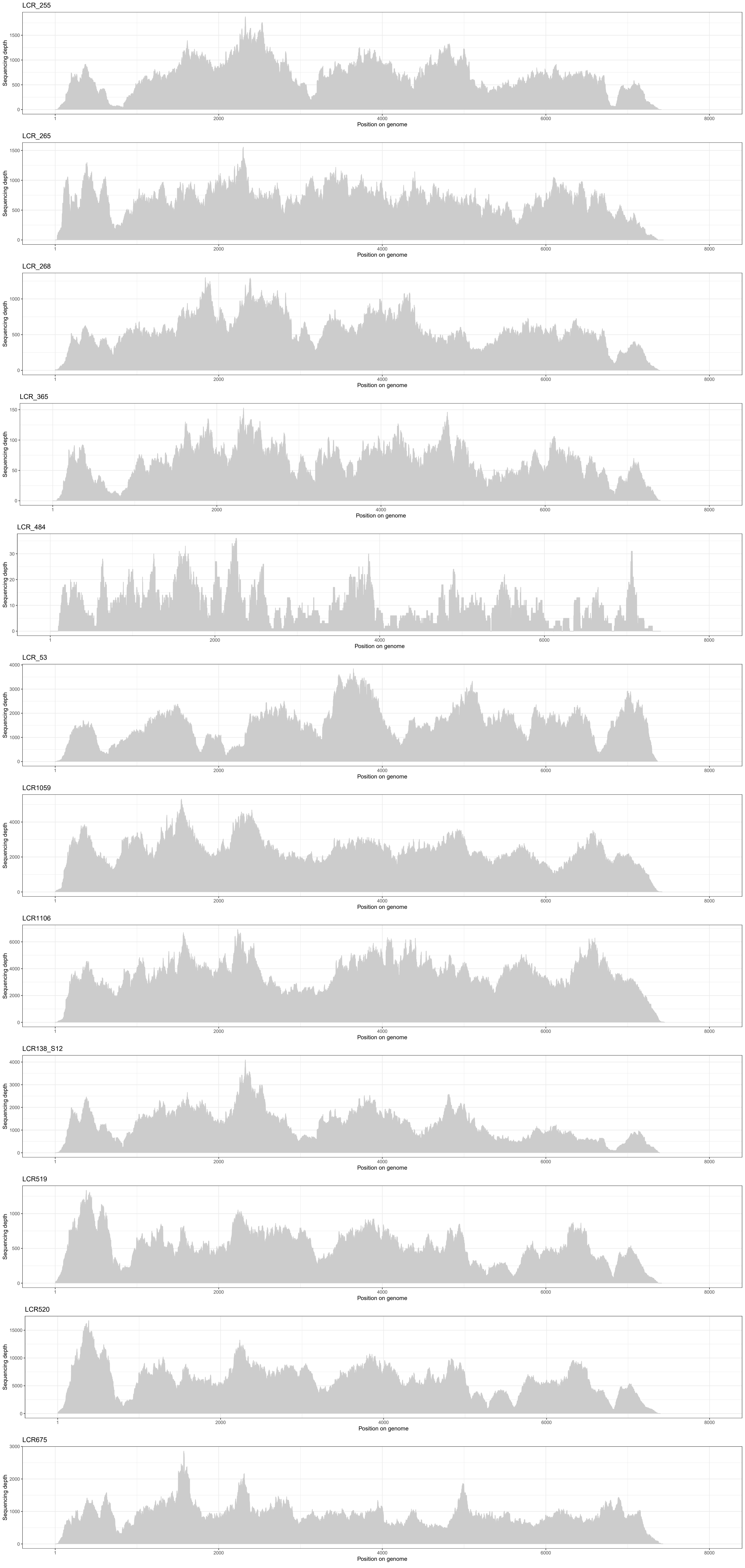

Supplement: Supplementary file 2 — Supplementary Figure S1. [file 41598_2021_1053_MOESM2_ESM.pdf]

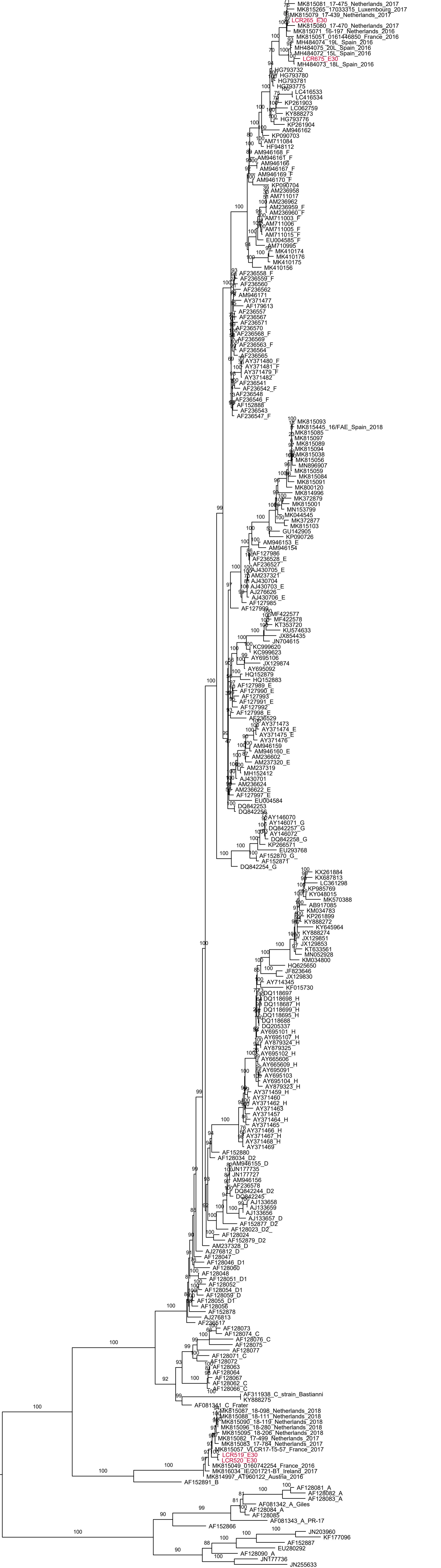

Supplement: Supplementary file 3 — Supplementary Figure S2. [file 41598_2021_1053_MOESM3_ESM.pdf]

A)

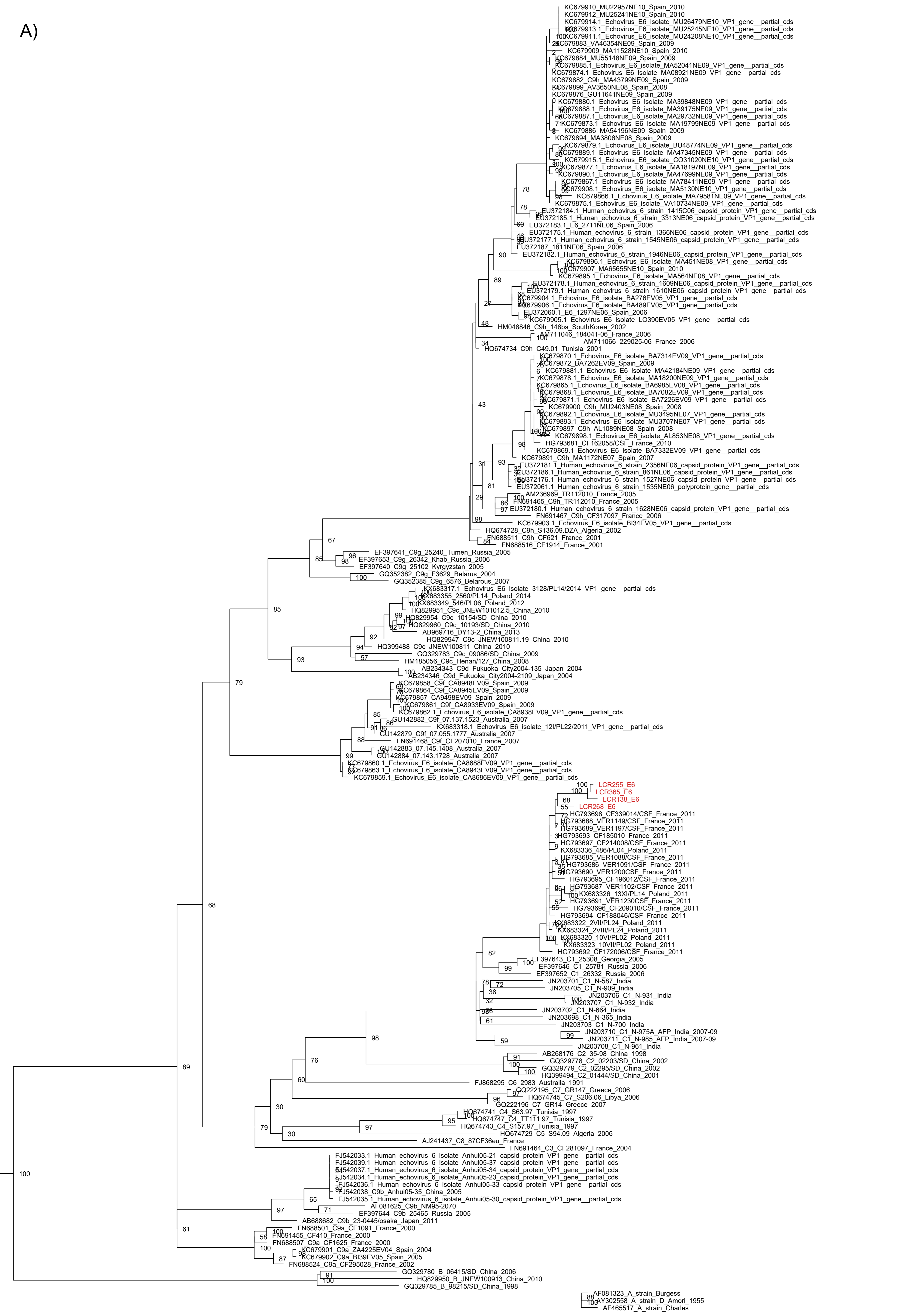

0.04

Supplement: Supplementary file 5 — Supplementary Figure S4. [file 41598_2021_1053_MOESM5_ESM.pdf]

B)

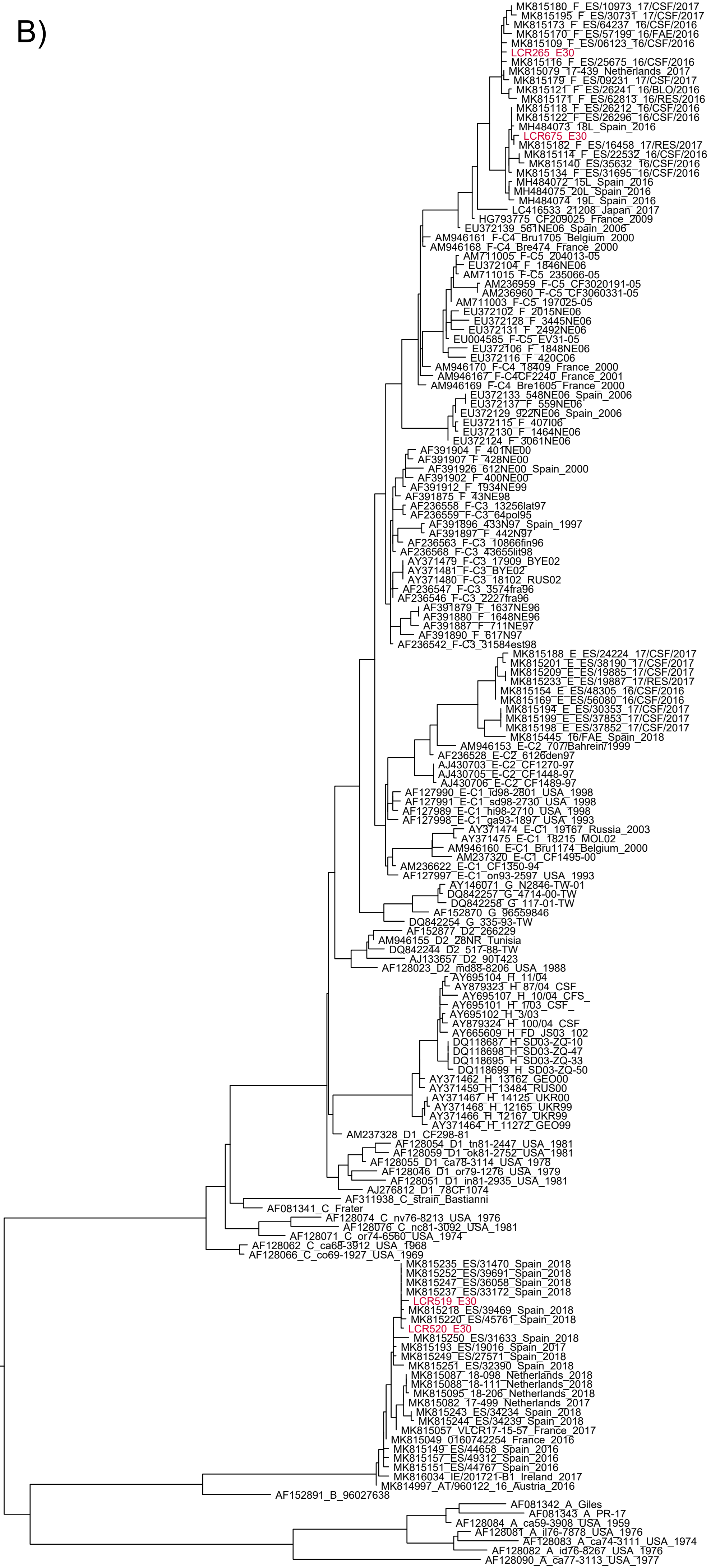

0.04

Supplement: Supplementary file 6 — Supplementary Figure S4. [file 41598_2021_1053_MOESM6_ESM.pdf]

C)

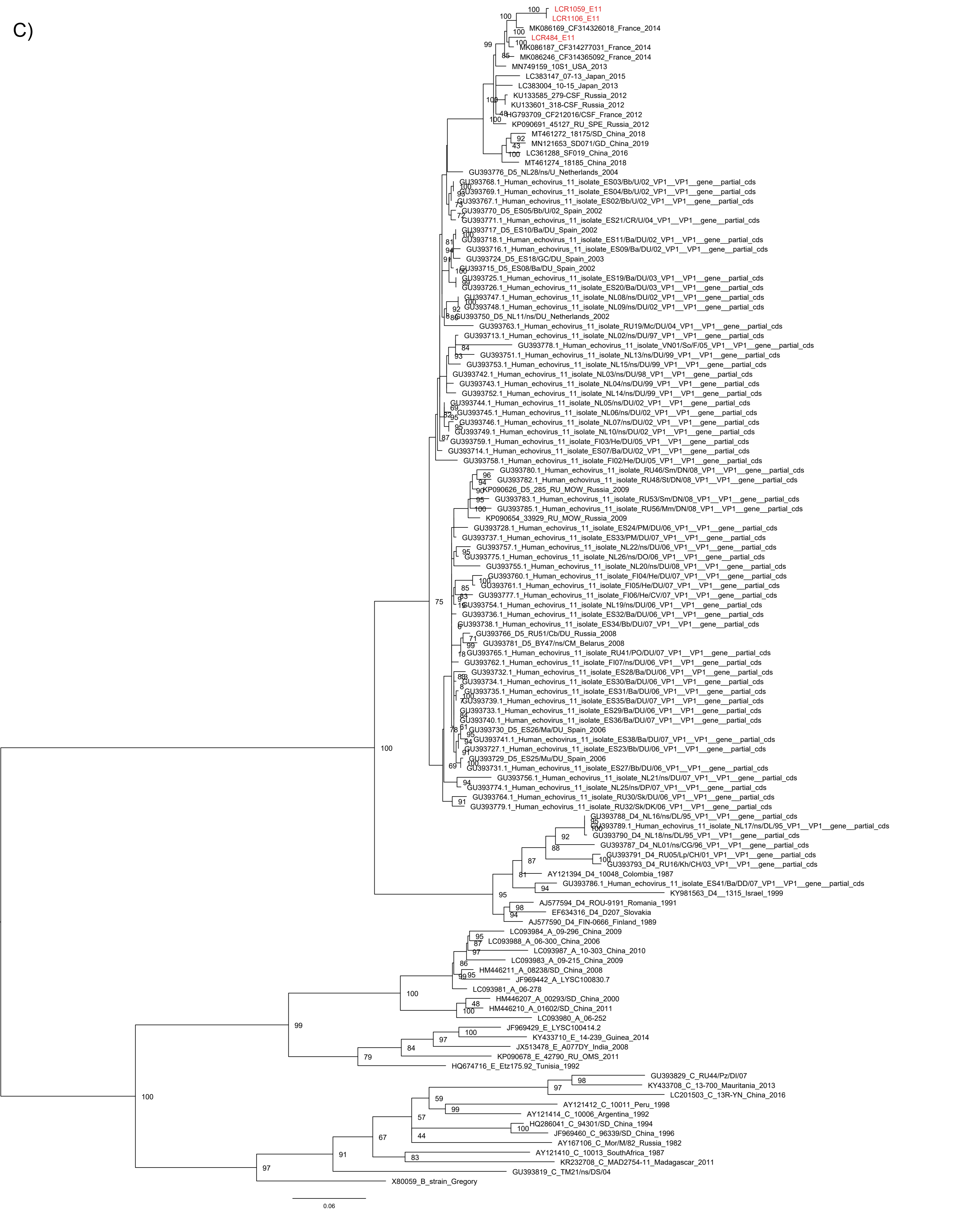

Supplement: Supplementary file 7 — Supplementary Figure S4. [file 41598_2021_1053_MOESM7_ESM.pdf]

D)

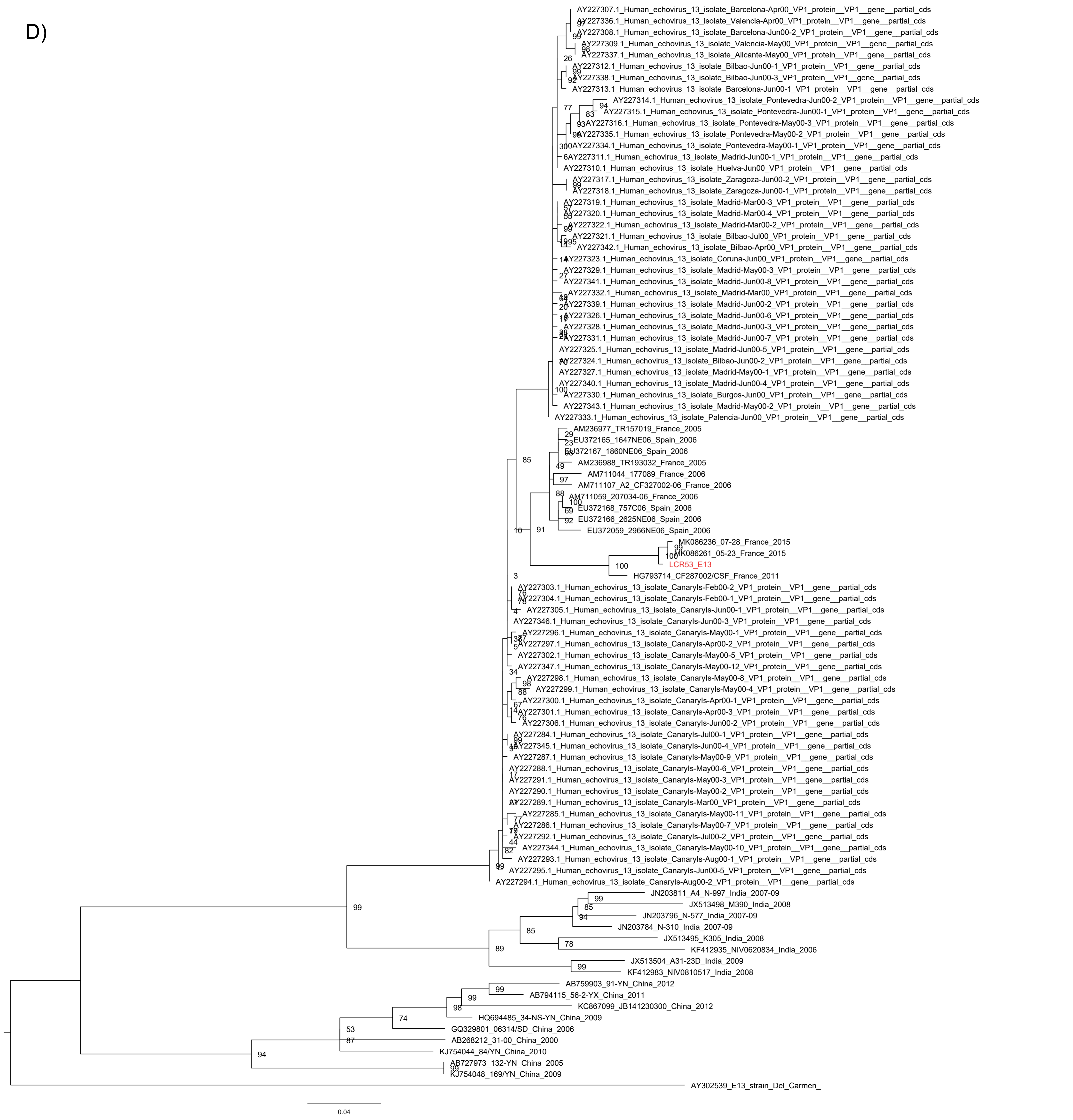

Supplement: Supplementary file 8 — Supplementary Figure S4. [file 41598_2021_1053_MOESM8_ESM.pdf]
