# Supplementary material for: Genomic surveillance of enterovirus associated with aseptic meningitis cases in southern Spain, 2015–2018
Source: Sci Rep. 2021 Nov 2;11:21523. doi: 10.1038/s41598-021-01053-4 (PMC8564535; doi:10.1038/s41598-021-01053-4)
Supplement: Supplementary file 4 — Supplementary Figure S3. [file 41598_2021_1053_MOESM4_ESM.pdf]

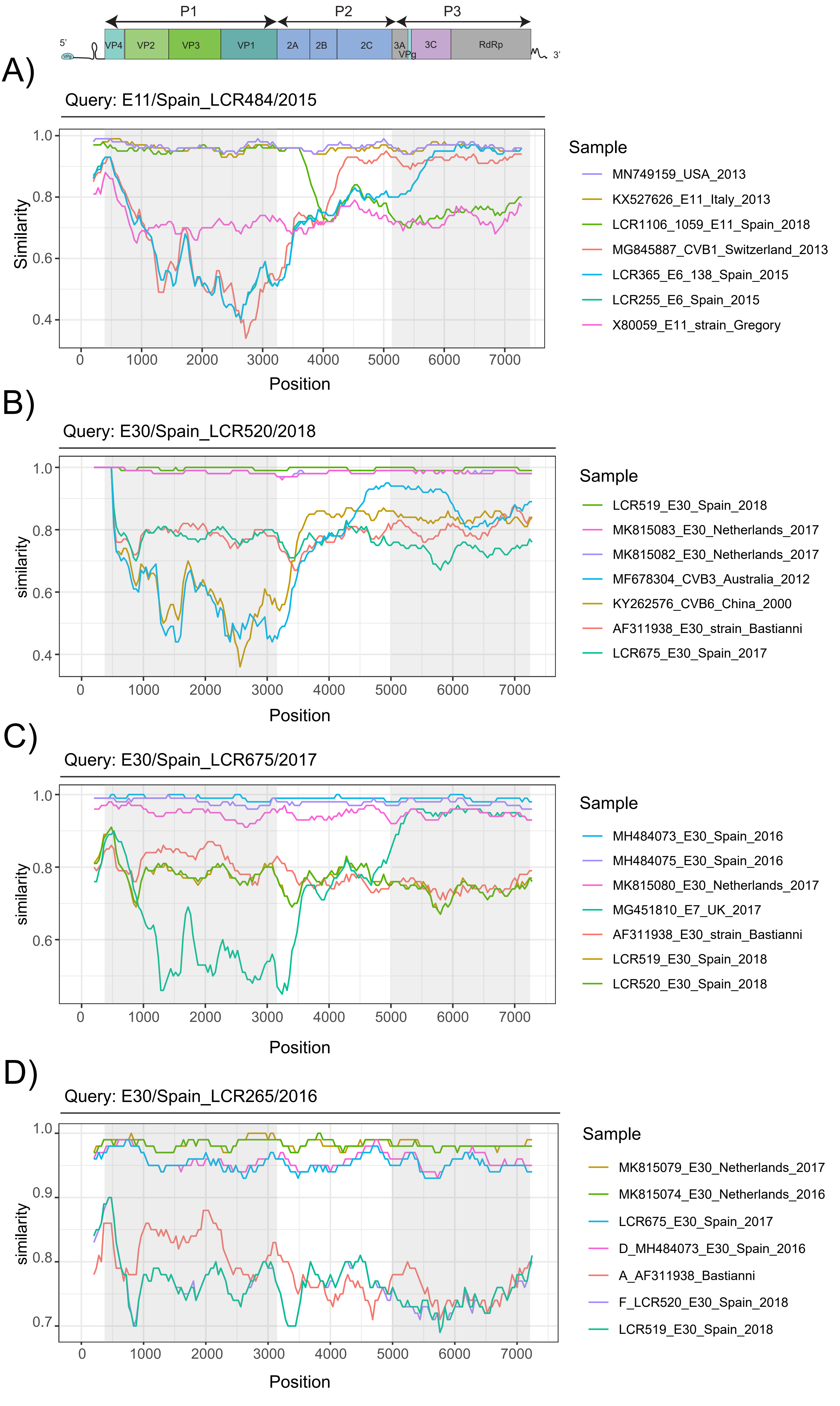
P1
P2
P3
5'
VPg
VP4
VP2
VP3
VP1
2A
2B
2C
3A
VPg
3C
RdRp
3'
A)
Query: E11/Spain_LCR484/2015
Similarity
Position
Sample
MN749159_USA_2013
KX527626_E11_Italy_2013
LCR1106_1059_E11_Spain_2018
MG845887_CVB1_Switzerland_2013
LCR365_E6_138_Spain_2015
LCR255_E6_Spain_2015
X80059_E11_strain_Gregory
B)
Query: E30/Spain_LCR520/2018
similarity
Position
Sample
LCR519_E30_Spain_2018
MK815083_E30_Netherlands_2017
MK815082_E30_Netherlands_2017
MF678304_CVB3_Australia_2012
KY262576_CVB6_China_2000
AF311938_E30_strain_Bastianni
LCR675_E30_Spain_2017
C)
Query: E30/Spain_LCR675/2017
similarity
Position
Sample
MH484073_E30_Spain_2016
MH484075_E30_Spain_2016
MK815080_E30_Netherlands_2017
MG451810_E7_UK_2017
AF311938_E30_strain_Bastianni
LCR519_E30_Spain_2018
LCR520_E30_Spain_2018
D)
Query: E30/Spain_LCR265/2016
similarity
Position
Sample
MK815079_E30_Netherlands_2017
MK815074_E30_Netherlands_2016
LCR675_E30_Spain_2017
D_MH484073_E30_Spain_2016
A_AF311938_Bastianni
F_LCR520_E30_Spain_2018
LCR519_E30_Spain_2018
